# Organoids to Model Tumor Microenvironment in Progression of Pathogenesis and Treatment Resistance in Glioblastoma Multiforme

**DOI:** 10.3390/brainsci16050531

**Published:** 2026-05-18

**Authors:** Pranav Kalaga, Swapan K. Ray

**Affiliations:** Department of Pathology, Microbiology, and Immunology, University of South Carolina School of Medicine, 6439 Garners Ferry Road, Columbia, SC 29209, USA

**Keywords:** glioblastoma multiforme (GBM), tumor microenvironment (TME), treatment resistance, GBM organoids, cerebral organoid co-culture models, organoid-on-a-chip models

## Abstract

Glioblastoma multiforme (GBM) remains the most aggressive and therapeutically intractable primary brain tumor, with many patients experiencing rapid relapse despite maximal surgical resection followed by standard chemoradiation. This persistent failure reflects the convergence of profound tumor-intrinsic genetic heterogeneity and a highly dynamic, spatially structured, and immunosuppressive tumor microenvironment (TME). Together, these forces create strong selective pressures that fuel tumor evolution, intratumoral diversity, phenotype plasticity, diffuse invasion, and robust resistance to therapy. The TME of GBM is orchestrated through a complex interplay between diverse cellular constituents, including tumor-associated macrophages, reactive astrocytes, endothelial cells, pericytes, and GBM stem cells, and non-cellular components such as extracellular matrix remodeling, hypoxia, metabolic and nutrient gradients, and spatially patterned cytokine and chemokine signaling networks. Additionally, heterogeneity in blood–brain barrier (BBB) and blood–tumor barrier (BTB) complicates drug delivery and immune surveillance, reinforcing therapeutic resistance and regional tumor adaptation. Conventional two-dimensional cell cultures and animal models fail to sufficiently capture these multiscale, patient-specific interactions, limiting their translational predictive power. In this narrative review, we synthesize recent advances in GBM organoid technologies as physiologically relevant, three-dimensional platforms that more faithfully recapitulate TME for driving tumor evolution and treatment resistance. We compare complementary organoid strategies, including patient-derived GBM organoids that preserve native cytoarchitecture, cerebral organoid co-culture systems that reconstruct tumor–brain interactions, and advanced platforms incorporating immune and vascular features such as air–liquid interface cultures, microglia-enriched systems, and BBB/BTB-integrated models. Finally, we highlight emerging innovations such as spatial transcriptomics, organoid-on-a-chip systems, live imaging coupled with lineage tracing, genome engineering, and artificial intelligence integration that collectively position GBM organoids at the forefront of precision neuro-oncology, reproducing TME, enabling dynamic mapping of tumor evolution, and accelerating patient-specific therapeutic discovery.

## 1. Introduction

Glioblastoma multiforme (GBM) is an extremely aggressive and invasive brain tumor that has an incidence rate of 4.32 per 100,000 in the United States [1]. The median overall survival for patients with GBM is 10 months, and only 6.7% of patients survive past 2 years [2]. Emerging evidence suggests that recurrence-like changes after chemoradiotherapy may reflect either true tumor progression or pseudo-progression, and that true progression is associated not only with larger tumor volume but also with heightened systemic inflammation [3]. Furthermore, GBM presents an exceedingly difficult challenge to treat due to its highly invasive nature, intratumoral heterogeneity (ITH), and immune evasion. GBM cells are highly invasive since they can effectively colonize blood vessels and glial cells [4]. Almost 85% of brain tumors are GBM that have already permeated into the brain parenchyma at diagnosis [5]. ITH enables individual GBM cells within a tumor to express different genes, morphologies, and behaviors, making it difficult to deliver tailored therapies [6]. GBM has also been reported to inhibit the major histocompatibility complex I and II, and production of T cells, highlighting its dangerous immunosuppressive capabilities [7].

These troubling characteristics also face current challenges when it comes to the development of accurate GBM models. The most basic and prevalent type of GBM model is a two-dimensional (2D) in vitro cell culture [8]. Although this type of model is relatively easy to create and reproduce, it fails to accurately simulate the GBM tumor microenvironment (TME) and constrains the precise development of ITH [9]. There is a growing consensus that 2D in vitro models of GBM are unreliable, as many anticancer agents assessed on these 2D models fail to successfully transition into clinical implementation [10]. Another popular GBM model uses mice that are xenotransplanted with GBM cells originating from a cell culture or a tumor in a patient [10]. Both animal models with the cell culture and patient-derived xenografts have demonstrated accuracy in replicating the TME and producing ITH, like what is seen in patients with GBM [10,11]. However, some concerns with these xenograft models are that they require an extremely elevated level of skill and experience to successfully grow and that patient-derived xenograft models take a longer time to develop than cell culture-derived xenograft models [12,13]. Furthermore, both xenograft models require that mice be immunodeficient, clouding any conclusions about the immunosuppressive nature of GBM and its interactions with the immune system [14].

Given the current limitations of the 2D in vitro and mouse xenograft models, GBM organoids have emerged as relatively accurate 3D models that present a new opportunity in GBM modeling [15]. Organoids are the 3D in vitro models that are usually grown from induced pluripotent stem cells (iPSCs) or human embryonic stem cells (hESCs) in the presence of an extracellular matrix (ECM), growth factors, and other controlled environmental conditions [16]. After undergoing differentiation, the iPSCs or hESCs transform into a cerebral organoid that mirrors an embryonic brain, including its microglia, signaling pathways, and vascular structure [17]. After the cerebral organoid has developed, it can be manipulated through genetic modification, co-cultures, and other methods to produce GBM organoids [18,19]. Furthermore, numerous studies have shown that GBM organoids successfully reproduce TME, ITH, and tumor invasiveness, making them relatively ideal models for studies related to GBM [20,21,22]. A crucial feature of GBM organoids is that they uniquely recreate environmental pressures that shape tumor evolution and therapy resistance. The 2D cell cultures and mice xenografts are limited in their ability to simulate the environmental pressures that drive GBM cells in human brains to develop ITH and immunosuppressive abilities [23]. Through time-lapse imaging, GBM organoids enable an up-close analysis of the mechanisms of GBM cell invasion patterns, the TME, and interactions between GBM cells and healthy brain cells [24]. In addition to organoids, spheroids represent another class of 3D in vitro models that function similarly to organoids but are relatively simpler models that present with less structural complexity [25].

The literature search for preparation of this narrative review included PubMed, Google, and the National Institutes of Health (NIH) Clinical Trials database. Keywords in the search criteria included GBM, organoids, TME, and artificial intelligence (AI). Additionally, some relevant literature not sourced from the search criteria was included to provide context and support our outcomes from the literature. In this narrative review article, we integrated TME modeling with evolutionary dynamics in GBM organoid systems by examining how organoid-based platforms recapitulate key selective pressures such as hypoxia, nutrient gradients, cellular interactions, and immune modulation. By synthesizing recent advances in GBM organoid technology, we highlight how these models can enable the study of tumor evolution, ITH, immunosuppression, and therapy resistance in a controlled yet physiologically relevant context. Ultimately, this integrated perspective underscores the potential of GBM organoids to bridge the gap between experimental modeling and clinical translation, facilitating the development of more effective and enduring therapeutics for the management of GBM.

## 2. TME of GBM and Key Drivers of Tumor Evolution and Treatment Resistance

### 2.1. Cellular Components in the TME of GBM

GBM is characterized by an extraordinarily complex and dynamic TME that actively shapes tumor evolution, ITH, and therapeutic failure. Rather than serving as a passive scaffold, the TME functions as an evolving ecosystem in which cellular components exert selective pressures, promote phenotypic plasticity, and enable adaptive resistance to various treatments [26]. Among the most influential constituents in the TME of GBM are tumor-associated macrophages (TAMs), reactive astrocytes, vascular cells, including endothelial cells and pericytes, and GBM stem cells (GSCs) [27]. Accurate and predictive modeling of GBM progression and therapy response requires explicit consideration of all these interacting cellular compartments.

TAMs represent one of the most abundant cell populations in GBM, often comprising up to half of the tumor mass [28]. These cells arise from both brain-resident microglia and bone marrow-derived monocytes and are actively recruited and reprogrammed by tumor-derived signals such as colony stimulating factor-1 (CSF-1), chemokine (C-C motif) ligand 2 (CCL2), and transforming growth factor-beta (TGF-β) [29,30]. Rather than mounting effective anti-tumor immune responses, TAMs adopt a GBM-specific, immunosuppressive phenotype that promotes tumor growth and invasion. Through the secretion of matrix metalloproteinases (MMPs), cytokines, and angiogenic factors, TAMs facilitate local invasion, vascular remodeling, and immune evasion [31]. Importantly, spatial and functional heterogeneity among TAM populations contributes directly to ITH by creating region-specific microenvironments that favor distinct GBM cell states [32]. Single-cell RNA sequencing (scRNA-seq) and spatial transcriptomic studies have linked TAM-rich regions to mesenchymal tumor phenotypes, which are associated with increased aggressiveness and poor prognosis [33,34,35]. Following therapy, TAMs often expand and further suppress immune surveillance, thereby facilitating tumor recurrence and resistance to chemotherapy [36].

Astrocytes, the most abundant glial cell type in the healthy brain, also play a critical role in shaping the GBM microenvironment through reactive astrogliosis, a proliferative state that supports GBM progression through a variety of avenues [37]. In response to tumor invasion, astrocytes undergo transcriptional and functional changes that transform them into active participants in tumor progression [38]. Reactive astrocytes secrete growth factors and cytokines that support GBM cell survival, proliferation, and invasive migration [39]. Direct cell-to-cell communication via gap junctions further enables astrocytes to transfer metabolic and survival signals to tumor cells, buffering them against therapeutic stress [40]. These interactions between astrocytes and GBM cells promote phenotypic plasticity, allowing GBM cells to transition between proliferative and invasive states depending on local conditions [41]. As a result, astrocyte-rich niches often harbor tumor cells that are more resistant to cytotoxic therapies [38].

The vascular compartment in the TME of GBM, composed primarily of endothelial cells and pericytes, is another major driver of tumor evolution and heterogeneity. GBM is hallmarked by aberrant vascular proliferation, driven by tumor-secreted angiogenic factors such as vascular endothelial growth factor (VEGF) [42]. Endothelial cells not only supply oxygen and nutrients but also secrete angiocrine growth factors (specialized molecules released from endothelial cells to line the blood vessels) that influence tumor cell angiogenesis through pathways such as Notch and nitric oxide signaling [43,44]. Pericytes, which arise through differentiation of GSCs, contribute to tumor vascular stabilization and further reinforce tumor–vascular interactions [45]. Heterogeneity within the vascular network of GBM generates spatial gradients of oxygenation and nutrient availability, creating hypoxic niches that promote genomic instability, selection for aggressive tumors, and maintenance of stem-like tumor states [46,47,48]. These same features also limit drug delivery and reduce the efficacy of radiotherapy, while adaptive responses to anti-angiogenic therapies can paradoxically enhance tumor invasiveness [49,50].

GSCs represent a subpopulation of tumor cells with self-renewal capacity and multilineage differentiation potential [51]. Experimental and genomic evidence suggest that GSCs may arise from multiple sources, including neural progenitor cells and glial progenitors such as oligodendrocyte-progenitor-like and astrocyte-like [52]. GSCs are localized to specialized microenvironmental niches where they receive niche-derived signals that maintain stemness and survival. GSCs are preferentially enriched in hypoxic and perivascular niches, where selective pressures favor traits such as metabolic flexibility, enhanced DNA damage and repair, and resistance to apoptotic cell death [48]. Importantly, the GSC state is increasingly recognized as plastic rather than fixed, with non-stem GBM cells capable of acquiring stem-like properties through epigenetic reprogramming [53]. This bidirectional pathway enables the dynamic evolution of GSC populations in response to stresses like chemotherapy. GSCs have three main attributes that elevate therapeutic resistance: their quiescent nature, enhanced DNA damage and repair capacity, and elevated expression of drug efflux transporters, avoiding induction of regulated cell death mechanisms such as apoptosis and ferroptosis [54,55,56]. The survival of GSCs via induction of autophagy following treatment is indicative of tumor recurrence [57]. Cell types in the TME thus have profound impacts on the survival of GBM (Table 1).

**Figure 1:**
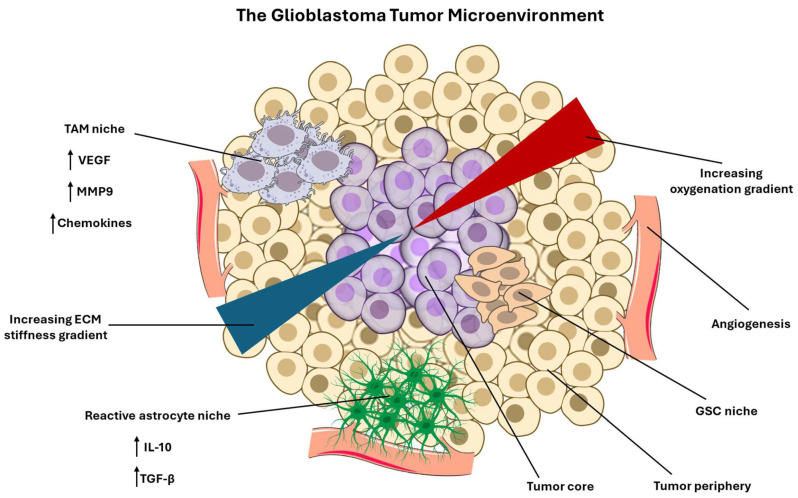
Cellular and non-cellular components in the TME of GBM. At the center of the tumor, the core contains a necrotic hub with hypoxic conditions and low stiffness. The tumor periphery has greater oxygenation and stiffness, which promotes more invasive phenotypes. A common feature of GBM is aberrant angiogenesis, allowing the tumor to take control of blood vessel formation to dysregulate blood flow. There are several cellular niches in the TME of GBM: TAMs, reactive astrocytes, and GSCs. The formation of niches allows for the consolidation of harmful cell populations, enabling greater treatment resistance and recurrence of the tumor.

Collectively, these cellular constituents in the TME of GBM form an interconnected and adaptive ecosystem that shapes tumor evolutionary dynamics. TAMs, astrocytes, and vascular cells establish spatially structured niches that modulate selective pressures while GSCs exploit these niches to preserve heterogeneity and confer resistance to treatments. Modeling GBM progression and treatment response requires frameworks that move beyond basic tumor properties to incorporate TME dynamics, spatial heterogeneity, and cell–cell interactions. Integrative models that capture these features will be essential for understanding therapy resistance and for designing therapeutic strategies capable of disrupting the ecological support systems that sustain GBM malignancy. Various cellular and non-cellular components in the TME of GBM act in a symphony to promote its progressive pathogenesis and confer treatment resistance (Figure 1).

### 2.2. Non-Cellular Components of the TME of GBM

GBM progression and recurrence are increasingly understood as outcomes of evolutionary dynamics within a spatially structured TME, in which non-cellular features such as biophysical constraints, oxygen and nutrient availability, inflammatory mediators, and vasculature are selection pressures that favor specific tumor and immune phenotypes [68]. GBM is associated with extensive ECM remodeling characterized by increased matrix density and aberrant expression of ECM components, including hyaluronic acid, tenascin-C, fibronectin, proteoglycans, and MMPs [69]. ECM stiffness is spatially heterogeneous, with evidence consistent with a gradient in which the core is relatively softer while stiffness increases toward tumor edges, a pattern that may condition invasive behavior through persistent mechanical memory [70]. Tumor cells directly remodel the ECM by altering synthesis and organization of matrix components and by exerting forces that reorganize the surrounding matrix [71]. Specifically, they transduce stiffness through pathways like integrin/focal adhesion kinase (FAK) and Yes-associated protein and transcriptional coactivator with PDZ (the first letters of the first three proteins) domain or binding motif (YAP/TAZ) [72]. GBM-specific work highlights Piezo1, a transmembrane ion channel, as a mechanosensor linking matrix stiffness to pro-invasive and mesenchymal-like states via YAP/TAZ activity [73]. TAMs contribute to ECM remodeling through the secretion of proteases that degrade and restructure ECM, potentially expanding permissive invasion paths and altering immune trafficking through changes in membrane integrity [74]. Endothelial cells and perivascular populations shape the perivascular ECM and, in doing so, regulate both tumor cell migration along the vessels and leukocyte transmigration [46]. Spatial analysis of the recurring GBM demonstrates that the ‘texture’ of ECM can be predictive of single-cell genetic traits, providing evidence that the architecture of ECM is not merely a consequence of tumor growth but can actively structure tumor evolution and environment [75].

GBM commonly exhibits profound hypoxia and necrosis arising from abnormal vasculature, high metabolic demand, and perfusion defects, generating steep oxygen and nutrient gradients that function as strong selection pressures for progressive tumor growth [47]. Current literature emphasizes hypoxia and the resulting necrosis as a driver of tumor evolution through metabolic adaptation, ECM remodeling, and immune evasion [76]. Tumor cells both create and respond to hypoxia by upregulating stress-response programs that support survival under low oxygen and nutrient gradients, contributing to treatment resistance and recurrence [77]. Endothelial cells and pericytes contribute by establishing a vasculature that is frequently abnormal in structure and function, enabling spatial heterogeneity in perfusion and thereby maintaining persistent hypoxic gradients [78]. TAMs are selectively recruited and polarized within hypoxic and perinecrotic regions [79]. Mechanistic evidence identifies a hypoxia-conditioned macrophage state in perinecrotic niches that can further promote vascular dysfunction and permeability, illustrating how hypoxia remodels the TME of GBM [80].

Hypoxia promotes immunosuppression through multiple, partially redundant mechanisms. A key pathway is the accumulation of extracellular ATP in hypoxic environments, followed by signaling through the adenosine A2A receptor (A2AR) and A2BR, which are expressed on immune cells, thereby dampening cytotoxic function and supporting immunosuppressive programs [81]. Hypoxic compartments are also associated with recruitment and polarization of immunosuppressive TAMs and enrichment of immune-excluded niches, further limiting effective antitumor immunity [82]. Hypoxia enforces regional specialization. Hypoxic niches select for cells optimized for oxidative stress tolerance, altered nutrient utilization, and invasive escape behaviors, while perivascular oxygenated regions may support alternative growth and stemness programs [83]. This compartmentalization increases ITH by enabling the coexistence of multiple phenotypes within a single lesion [84]. Hypoxia also drives plasticity by promoting reversible transcriptional shifts, such as stress-adaptive and invasion-associated changes, and by initiating ECM remodeling that further reshapes the tumor landscape [85,86].

In GBM, cytokine and chemokine expression form spatially heterogeneous signaling niches that regulate immune recruitment, immune differentiation, and tumor cell state transitions [87]. Central pathways include IL-6–STAT3 signaling, TGF-β-dependent immune suppression programs, and chemokine axes like CCL2 and CCL7 that coordinate myeloid recruitment and therapy resistance [88,89,90]. Tumor cells actively shape cytokine landscapes. Studies demonstrated that GBM-derived IL-6 is necessary and sufficient for myeloid programmed death-ligand 1 (PD-L1) induction through a STAT3-dependent mechanism, linking tumor-secreted cytokines to immune checkpoint reinforcement and CD8-dependent immunosuppression in vivo [91]. Activated STAT3 is intertwined with cytokine networks, such as IL-10 and TGF-β, which maintain immunosuppressive populations [92]. Perivascular cells have emerged as additional organizers of cytokine and chemokine signaling. Transcriptomic profiling of GBM stroma implicates perivascular fibroblasts in overexpression of chemotactic factors associated with TAM recruitment and cancer stemness and links these populations to poor outcomes and reduced response to immune checkpoint therapies [93]. Furthermore, research suggests that CCL2 secreted by cancer-associated fibroblasts strengthens TMZ resistance in GBM, illustrating how stromal-derived factors can generate therapy-linked selection pressures [94]. Reactive astrocytes contribute to inflammatory signaling and tumor progression, participating in local feedback circuits that can shape cytokine distributions and immune accessibility [95]. Cytokine signaling also shapes tumor cell state transitions and contributes to phenotype plasticity. Studies identified a STAT3-controlled chitinase-3-like protein 1 (CHI3L1)/secreted phosphoprotein 1 (SPP1) positive feedback loop involving tumor cells and TAMs that promote proneural-to-mesenchymal transition and reflect immunosuppressive characteristics, directly connecting cytokine-associated transcriptional programs to regional heterogeneity and aggressive switching of phenotype [96]. Spatially patterned cytokine fields function as environmental drivers of ITH by enabling different niches to maintain distinct immune ecologies and tumor transcriptional states, while remaining dynamically interconvertible under changing pressures [97].

Drug and immune access to GBM is constrained by the blood–brain-barrier (BBB) and its tumor-modified counterpart, the blood–tumor-barrier (BTB), which form a spatially heterogeneous and actively regulated interface governing transport [98]. Endothelial cells define tight junction architecture and express active efflux transport systems. BBB efflux by the transporters ATP-binding cassette B1 (ABCB1) and ABCG2 is widely recognized as a major determinant limiting the effectiveness of many anti-tumor agents [99]. Pericytes and astrocytes contribute to BBB maintenance and vascular stability, and their remodeling can alter BBB permeability and perfusion [100]. From an evolutionary perspective, BBB and BTB heterogeneity create spatially patterned regions of drug exposure, generating niches that protect tumor compartments from therapeutic concentrations and imposing strong selection for phenotypes promoting recurrence [101,102]. Since barrier restrictiveness also constrains immune cell trafficking and the distribution of immunotherapeutics, BBB and BTB heterogeneity reinforce immunosuppression [103].

Across these axes, GBM exhibits a TME in which selection pressures are spatially segregated yet mechanistically coupled. Hypoxia promotes immunosuppressive metabolites and myeloid recruitment while also shaping vascular dysfunction and ECM remodeling, thereby feeding into both barrier heterogeneity and the mechanical landscape. ECM stiffness not only biases invasion and metabolic programs but can also reduce immune response, providing a direct link between TME structure and immunosuppression. Cytokine and chemokine gradients further consolidate immunosuppressive niches while driving tumor state transitions that expand the repertoire of stress-adapted phenotypes available for selection. Collectively, available evidence supports a model in which complex TME of GBM and therapeutic resistance are not solely a function of cell-intrinsic genetics, but also a consequence of non-cellular selection pressures, which are actively engineered by the tumor–stroma–immune interactions.

## 3. Organoids for Modeling the TME of GBM

### 3.1. Patient-Derived GBM Organoids Preserve Tumor-Intrinsic Microenvironmental Gradients and Heterogeneity with Minimal Exogenous Manipulation

A foundational approach for modeling GBM-relevant microenvironmental cues is the generation of patient-derived GBM organoids (PDGBOs) directly from resected tumor tissue with minimal processing. An efficient method for the development of PDGBOs is described by Jacob and colleagues [104]. Freshly resected patient-derived specimens are rapidly transferred to cold buffered solution, de-bulked, and dissected into pieces between 0.5 and 1 mm rather than enzymatically dissociated to single cells. Following red blood cell lysis and serial washes, tumor fragments are cultured in suspension in a “GBO medium” that is intentionally formulated to reduce clonal selection pressures, specifically avoiding serum and commonly used mitogens such as epidermal growth factor (EGF) and basic fibroblast growth factor (bFGF), as well as avoiding embedding in Matrigel. To promote oxygenation and diffusion while maintaining 3D organization, fragments are maintained in ultra-low attachment conditions with continuous agitation via orbital shaking, regular medium exchanges, and careful handling [104].

This explant-style strategy is consequential for genomic fidelity and the retention of ITH [105]. PDGBOs can preserve the histological diversity of the patient tumor over relatively short culture windows, making them well-suited for studies that require patient-specific responses [106]. In practice, genomic fidelity is commonly monitored by comparing organoid and parental tumor profiles across multiple layers, such as targeted sequencing for driver variants, copy-number analyses, and methylation state mapping paired with immunohistochemical validation of hallmark features of GBM. This is applied in the PDGBO approach reported by Verduin and colleagues [21]. Longitudinal sequencing across months of culture demonstrated high retention of variants over time, while single-cell karyotype-based measurements supported preservation of genetic heterogeneity rather than convergence to a dominant clone. Importantly, these PDGBOs displayed differential TMZ sensitivity that corresponded with O6-methylguanine-DNA methyltransferase (MGMT) promoter methylation status, underscoring how genomic preservation enables clinically relevant phenotyping in vitro [21]. The PDGBO framework has also been explicitly positioned for translational deployment, including biobanking and therapy testing by Jacob and colleagues. They used PDGBOs to track GBM mutational profiles with drug sensitivity and model chimeric antigen receptor T (CAR T) cell activity in a patient-specific context [107].

Importantly, even without adding exogenous stromal components, PDGBOs can preserve several TME-like features that emerge from 3D geometry and tumor-intrinsic programs [108]. These include microenvironmental gradients that drive spatial patterning of proliferation and stress responses, and the retention of local cell–cell interactions present in the patient tumor [109]. As a result, PDGBOs often provide a baseline for TME-relevant phenotypes such as growth, invasion potential, and therapy resistance against which more complex microenvironmental reconstructions can be compared [110]. This is particularly clear in GBM organoid paradigms explicitly designed to model a pre-angiogenic and pre-immune infiltration phase, where the absence of vascular and immune contaminants is treated as an advantage for isolating tumor-intrinsic programs. In a growth factor–free PDGBO organoid model, Watanabe and colleagues used transcriptomic and histologic characterization to show that organoids develop compartmentalized proliferative and stressed regions consistent with diffusion-limited growth and display measurable hypoxia gradients [111]. Notably, despite lacking immune populations, PDGBOs exhibited enrichment of immune-associated programs like cytokine secretion and antigen presentation signatures, supporting the broader concept that some immunomodulatory phenotypes in GBM are tumor-intrinsic and can be examined in the absence of various immune components [112].

### 3.2. Microglia- and Immune-Enhanced Organoids Enable Mechanistic Dissection of Neuroimmune Crosstalk and Immunotherapy Response

Since GBM is strongly shaped by immune suppression and myeloid-driven remodeling, a major direction in organoid engineering has been the incorporation of immune system components such as macrophages and even patient-derived lymphocytes. A key conceptual distinction is whether immune cells are retained from the original tissue, introduced as defined exogenous populations, or generated endogenously within the organoid system. One strategy to preserve endogenous immune populations is the air–liquid interface (ALI) organoid method, which was developed for patient-derived organoids that preserve immune cells within intact tumor architecture [113]. Neal and colleagues demonstrated that ALI cultures can propagate tumor organoids while retaining native immune components such as T cells, B cells, natural killer (NK) cells, and myeloid populations and can preserve immune features and model checkpoint blockade responses [114]. It has been revealed that preserving immune content in organoid models is most feasible when tissue architecture is minimally disrupted, and culture conditions support short-to-intermediate term survival of multiple lineages. Although this work was not GBM-specific, it provided a pathway to retaining immune populations in tumor organoid cultures.

A second, increasingly common approach is to add defined microglia populations to brain organoids. Multiple protocols now exist to generate human microglia-like cells from iPSCs and to integrate them into organoids. Abud and colleagues described a defined differentiation route to produce iPSC-derived microglia-like cells and integrate them with cerebral organoids, providing a fundamental framework for co-culture experiments [115]. Separately, microglia can also arise within cerebral organoid protocols, where mesodermal progenitors give rise to organoid-grown microglia, as shown by Ormel and colleagues [116]. To improve control over microglia abundance and timing, Jiang and colleagues reported a co-culture framework in which primitive macrophage progenitors were combined with neural progenitors to yield microglia-containing brain organoids with a tunable microglia composition [117]. More recently, protocol refinements have emphasized the importance of the culture microenvironment itself. For example, microglia-ALI-cortical organoid approaches leverage an ALI configuration to support long-term microglial survival and detailed neuroimmune interactions, improving the accuracy of homeostatic versus activated microglial states in cerebral organoids [118].

**Figure 2:**
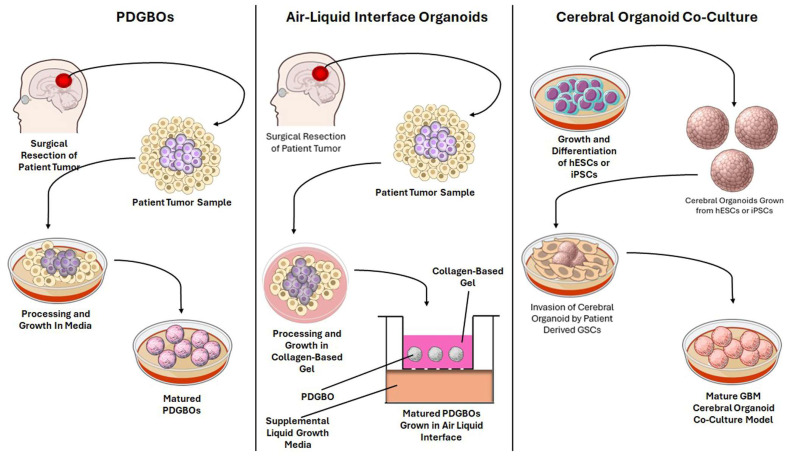
Methods of developing GBM organoids. PDGBOs are developed by resecting and isolating a tumor sample from the patient. This tumor sample is processed and grown in specific media, promoting the formation of PDGBOs. PDGBOs are especially useful since they can produce a more individualized model of a patient’s tumor, allowing for the development of patient-specific treatments. The air-liquid interface (ALI) method forms PDGBOs with a different workflow than traditional PDGBO methods. Resected patient-derived tumor samples are first grown in a collagen gel and then placed in a device that enables these tumor samples to be supplemented by liquid growth media, eventually forming mature PDGBOs. Cerebral organoid co-culture, also known as GLICO, is developed by co-culturing cerebral organoids with patient-derived GSCs. First, the cerebral organoid is developed from either hESCs or iPSCs. Then, patient-derived GSCs are introduced to the cerebral organoid, enabling the invasion of the GSCs. Once finished, the GSCs have completely invaded the cerebral organoid and formed a tumor.

Three different approaches—PDGBOs, ALI, and glioma cerebral organoid (GLICO)—are represented for modeling the TME of GBM in the laboratory settings (Figure 2).

A complementary immune-enhancement direction uses autologous tumor–brain organoid platforms to model patient-specific immune responses. Linkous and colleagues established an influential paradigm for analyzing GBM–brain organoid interactions called the GLICO model by seeding patient-derived GSCs onto human cerebral organoids and allowing them to infiltrate and expand within the host tissue. Methodologically, GSCs are first expanded in growth-factor-containing neural basal medium and then co-cultured by plating defined numbers of labeled tumor cells onto individual cerebral organoids for an initial static attachment period, followed by washing and transfer to long-term differentiation-like conditions on an orbital shaker to support sustained co-culture and invasion [19]. Azzarelli and colleagues detailed similar practical co-culture steps, including organoid quality control, fluorescent labeling of GSCs, and optimized co-culture conditions, aimed at preserving the heterogeneity fate of tumor cells as GSCs integrate into cerebral organoids [119]. Building on these immune- and microglia-enhanced platforms, the human organoid tumor transplantation (HOTT) framework leverages cerebral organoid and GBM cell co-culture to interrogate how neural microenvironmental cues modulate GBM cell states, emphasizing neural cell-enriched TME behavior [120]. Extending this concept, Baisiwala and colleagues introduced an autologous tumor–immune organoid platform called immune HOTT (iHOTT) designed to preserve patient-specific tumor–immune interactions, track T cell clonal responses, and mirror differential responses to PD-1 blockade [121]. While implementation may vary, these systems generally involve generating human cerebral organoids from pluripotent stem cells, transplanting freshly dissociated patient tumor cells into the organoid host tissue, and introducing matched immune populations such as patient-derived peripheral blood mononuclear cells or tumor-associated immune cells under conditions that sustain viability long enough to capture early immune activation, exhaustion, and cytokine signaling paths. Conceptually, these platforms address a major gap in GBM modeling: the inability of tumor-only organoids to reproduce immunotherapy-relevant dynamics without external immune reconstitution [122].

### 3.3. Vascularized and BBB/BTB-Integrated Organoids Connect Tumor Growth to Neurovascular Interfaces and Transport Barriers

Vascular biology and BBB function are crucial to GBM evolution and treatment response, yet they remain among the most difficult TME elements to reproduce in organoids [123]. Vascularized or BBB-integrated GBM organoid systems typically pursue one of two methodological directions. One direction aims to incorporate endothelial cells, often with pericyte and astrocyte support, into cerebral organoids to create microvascular-like networks that are structurally present and, in the best cases, perfusable [124]. The second direction uses microfluidic BBB devices in which engineered vessels and barrier compartments can be perfused and assessed for permeability, thereby enabling a controlled interface between a perfusion channel and an organoid-containing compartment [125]. Vascularized organized approaches attempt to incorporate endothelial elements into tumor constructs in a way that is less device-dependent but still captures vascular crosstalk. Co-culture of endothelial cells and cerebral organoids in the presence of growth factors can yield 3D structures with spatial organization and vascularization, providing a functional vascularized and BBB-integrated organoid for subsequent GBM integration [126]. Tatla and colleagues described a vascularized GBM tumoroid model that integrates vascular elements to better approximate tumor–endothelial interactions, creating an experimental context in which angiocrine signaling and vascular-associated invasion patterns can be studied [127]. In parallel, Dao and colleagues have demonstrated that fusing brain and blood-vessel organoids can yield BBB-containing assembloids with molecular and functional barrier properties [128]. Assembloids are sophisticated 3D, lab-grown tissue models formed by fusing multiple, distinct organoids or cell types to mimic complex, multi-region interactions, particularly of the brain [129]. Translating this concept toward a BTB context, Zhuang and colleagues describe BTB organoids generated by co-culturing human brain microvascular endothelial cells, astrocytes, pericytes, and patient-derived GSCs. These researchers noted that BTB organoids exhibited significant aberrant neovascularization, and transcriptomic analysis revealed pronounced genomic alterations favoring pro-mesenchymal states and GSC heterogeneity [130]. Although BTB organoid efforts remain in the initial stages, these studies collectively indicate that vascularized and BBB/BTB-organoids can be tuned toward vascular- and barrier-like phenotypes in ways that are difficult to achieve in tumor-only organoids [131].

Microfluidic perfusion is often introduced to address a persistent limitation of larger organoids: diffusion-limited delivery of oxygen and nutrients and the absence of stress cues that regulate endothelial phenotypes [132]. Vascularized organoid-on-a-chip models emphasize that microfluidic channels can enable controlled perfusion, spatially patterned ECM interfaces, and better regulation of mechanical cues, the features that are especially relevant when attempting to model vascular remodeling and drug transport across BBB- and BTB-like interfaces [133]. In one organoid-on-a-chip model, Straehla and colleagues have developed a predictive microfluidic model that recreates key aspects of GBM in a vascularized, BBB-relevant configuration and can be used to evaluate transport phenomena and therapeutic delivery constraints under controlled flow [134]. Finally, some vascularization strategies use engineered constructs that are organoid-adjacent, such as bioprinted microtissues, but still address TME-relevant vascular questions. Neufeld and colleagues have reported a perfusable 3D-bioprinted GBM model, demonstrating how engineered constructs can support vascular-like channels and controlled perfusion to maintain tumor viability while enabling defined delivery routes for drugs and other molecules of interest [135]. Such systems are especially valuable for elucidating how delivery kinetics, shear stress, and oxygenation shape tumor cell states, mechanistic axes that are often inadequately represented in static cell culture models [136]. All things considered, recent organoid models have indeed revolutionized our ability to accurately model GBM in a laboratory setting (Table 2).

### 3.4. ECM and Biophysical Microenvironment Engineering Reveals Invasion Programs Driven by GBM Mechanical Landscape

Beyond cellular composition, the TME of GBM is strongly constrained by ECM behavior and the mechanical landscape of brain and tumor tissues [70]. Organoids have therefore increasingly incorporated tunable biomaterials to interrogate transduction, migration, and invasion behaviors that are associated with ECM phenotypes [149]. Many ECM-focused organoid models use hyaluronic acid (HA)-based hydrogels with independently tunable mechanical properties. Sohrabi and Seidlits outline a protocol describing an ultraviolet (UV)-initiated thiol–ene hydrogel platform built on high molecular weight thiolated HA cross-linked with multifunctional poly-ethylene glycol (PEG) macromers, optionally functionalized with thiolated RGD (Arg-Gly-Asp tripeptide motif found in cell adhesion proteins like fibronectin) peptides to introduce adhesive cues [150]. The protocol details practical steps to fabricate hydrogel formulations, vary stiffness without changing total HA concentration by adjusting thiol concentrations, verify mechanical properties via rheometry, and culture uniformly sized, tunable patient-derived GBM spheroids, followed by downstream processing for immunofluorescence, RNA extraction for bulk sequencing, and protein analysis [150]. Rheometry is a technique used to measure the flow and deformation of materials (liquids, melts, suspensions, and solids) under applied stress, acting as a tool in understanding the state of 3D biological models [151]. It determines key properties like viscosity, elasticity, and plasticity, which are essential for controlling the ECM in organoids [152]. Because ECM composition and stiffness are key regulators of mesenchymal transition in GBM, rheometry is a crucial analytical tool for ECM-engineered organoids [72]. Another example of HA-hydrogel-integrated GBM organoids is provided by Liang and colleagues, who examined PDGBO growth within photocurable hydrogels and used transcriptomic profiling to assess how the hydrogel environment shapes organoid biology, highlighting that these PDGBOs retained the genomic and developmental profiles of their parent tumors [153].

Mechanically defined scaffolds have also been used to connect microenvironmental stiffness to functional phenotypic switching. Sohrabi and colleagues have linked stiffness to metabolic reprogramming and invasive behaviors, illustrating how engineered ECM spheroids can uncover GBM state transitions that depend on physical context rather than solely on genetics or soluble signaling [154]. Another innovation in ECM engineering is the use of bioprinting approaches that can reproducibly pattern complex microenvironments in models. Tang and colleagues reported a rapid digital light processing-based 3D bioprinting strategy to construct GBM microenvironment models in HA-rich matrices that incorporate patient-derived GSCs, macrophages, and astrocytes to study stemness, drug resistance, and immune interactions in a controlled ECM architecture [155]. Together, these ECM- and mechanics-focused platforms can connect organoid phenotypes to clinically relevant behaviors, particularly diffuse infiltration, TME remodeling, and adaptive resistance to treatments, by modeling the biophysical cues that GBM cells are likely to experience in vivo.

## 4. Evolutionary Dynamics of GBM Within Organoids

### 4.1. Heterogeneity Maintenance and Clonal Evolution

A central prerequisite for modeling GBM evolution is retention of ITH, including genetic factors like gene expression and non-genetic factors like cell-state diversity. Several organoid paradigms address this by either minimizing single-cell dissociation-induced selection or embedding tumor cells into brain-like microenvironments that enforce physiological conditions [106,107,156]. Complementary approaches include Matrigel- and growth-factor–supported tumoroid methods that support long-term expansion while producing diffusion-limited gradients. Hubert and colleagues described a 3D GBM organoid culture system that supported the growth of GBM organoids, emphasizing preservation of tumor architecture and microenvironmental gradients, notably hypoxia, linked to GSC heterogeneity [157]. In parallel, co-culture models such as GLICO provide a developmentally patterned neural context in which tumor cells migrate, invade, and form networks that resemble invasive behaviors in brain tissue and retain ITH [158]. More recently, Mangena and colleagues benchmarked cortical organoid co-culture systems against matched spheroid cultures and showed the GBM organoids to better preserve diversity of malignant transcriptional programs observed in patient tumors [159]. Methodologically, heterogeneity retention is increasingly validated using multi-layer readouts: histopathology, bulk genomics, and single-cell profiling to quantify tumor and non-tumor compartments, deduce copy-number variations, and track state proportions over time. For example, Krieger and colleagues developed a cortical organoid GBM system that explicitly leveraged scRNA-seq to highlight upregulation of malignant programs and transcriptional heterogeneity in organoids [160]. Similarly, Nicholson and colleagues have described how organoid co-culture systems grown long-term can be interrogated by single-cell multi-omic approaches combined with receptor–ligand modeling to identify microenvironmental remodeling events that influence clonal or stem-like expansion, an approach well suited to elucidate whether a change in tumor composition reflects intrinsic evolution versus extrinsic niche remodeling [161].

To map evolutionary paths, organoid systems are also compatible with longitudinal studies [162]. While direct barcoding and lineage tracing are not yet standardized across GBM organoids, the conceptual and technical foundation is established in GBM single-cell lineage studies and can be transferred to organoids by introducing heritable lentiviral barcodes followed by scRNA-seq readouts that link lineage identity to transcriptional state and ITH [52,163]. Lentiviral CellTagging provides an established framework for this purpose by introducing transcribed, heritable barcodes through a deactivated viral vector that can be recovered alongside single-cell transcriptomes, thereby linking lineage identity to cell state in the same assay [164]. CellTag-multi extends this concept across RNA and chromatin modalities, making it particularly attractive for GBM studies in which transcriptional and epigenetic plasticity may diverge under treatment [165]. These methods are therefore well suited for organoid-based evolutionary studies in which clone size, cell state, and state transitions must be resolved simultaneously. Although barcode-resolved lineage tracing is not yet standardized across GBM organoid studies, it is no longer merely conceptual. Fazzari and colleagues infected resected patient GBM cells with a CellTag barcode and transplanted these barcoded GBM cells into cortical organoids. Afterwards, GBM cells were harvested from the cortical organoids, and clonal analysis indicated differentiation of tumor progenitors into multiple mature GBM lineages, highlighting the heterogeneity within one tumor [166]. In another study, Fazzari and colleagues have used direct-from-patient lineage tracing in a GBM HOTT model to identify a progenitor population whose barcoded lineages generated both neural-like and mesenchymal/vascular-like malignant progeny, two states that are normally independent of each other [167]. These studies provide exactly the kind of organoid-based, lentiviral lineage-tracing evidence that was previously missing from the GBM organoid literature. For therapy-focused experiments, the most informative design is to barcode organoids at baseline and then sample pretreatment, early residual disease, and regrowth after TMZ treatment or radiation. This logic mirrored barcoded minimal-residual-disease studies in therapy-adapted GBM xenografts, where recurrence was shown to arise either from pre-existing high-fitness clones or from clones that acquired fitness under therapy [168]. Applied in organoids, the same longitudinal sampling framework may allow investigators to determine whether TMZ or radiation enriches pre-existing resistant clones, induces within-clone state transitions, or drives convergent evolution across many clones toward similar resistant states.

In practice, even without the use of barcoding and lineage tracing, niche-structured organoids support natural development that allows spontaneous spatial selection to emerge from diffusion constraints [169]. Labeling strategies such as live-organoid pimonidazole exposure to measure hypoxia and 5-ethynyl-2′-deoxyuridine (EdU) incorporation to measure proliferation, followed by sectioning and immunostaining, provide a practical route to quantify how clonal composition can evolve overtime and differ between niches [107]. Notably, EdU is a thymidine analog that gets incorporated into the newly synthesized DNA during S phase of the cell cycle, serving as a marker for actively dividing cells. Pimonidazole is a 2-nitroimidazole compound used for detecting hypoxic (low oxygen) regions in tissues, particularly tumors, by forming stable protein adducts at oxygen levels below 10 mmHg. It is administered intravenously or orally, with increased exposure (3–96 h) corresponding to higher detection of hypoxic cells. The important takeaway from this approach is that niche-specific selection and heterogeneity in organoids can arise without exogenous modification [170]. Long-term GLICO models show that as organoids age, chronic hypoxia and oxidative stress can remodel the microenvironment in ways that favor GSC expansion, implying that aging of the host tissue equivalent becomes an evolutionary variable in vitro [161]. Likewise, HOTT models highlight how defined neural microenvironments can bias GBM programs and behaviors, offering a path to experimentally knockdown and isolate which genes and signaling pathways impose selection on tumor states [120].

### 4.2. Cellular Plasticity and Phenotype Switching

GBM evolution is not driven solely by genetic diversification. Rapid phenotype switching allows tumor cells to adapt to fluctuating microenvironmental stresses and therapeutic pressures on timescales much shorter than those of clonal selection [171]. Organoid models offer a unique opportunity to observe such plasticity in a spatially structured, human-relevant context, where state transitions can be linked to specific niches and signaling cues. Aulestia and colleagues have shown that GSC initially coexists quiescently within developing organoids and later transitions into proliferative, invasive states as hypoxia and oxidative stress accumulate [172]. Hypoxia and oxidative stress are among the most consistently implicated drivers of GBM state transitions in organoid ecosystems. In GLICO models, single-cell and multi-omic analyses revealed that chronic hypoxia and oxidative stress upregulate ischemia-associated transcriptional programs in astrocytes and malignant cells, correlating with expansion of GSC and mesenchymal-like tumor states and accelerated tumor outgrowth upon exacerbation of oxidative stress [161]. Parallel work in GBM organoids by Shakya et al. has shown that hypoxic and perinecrotic regions are enriched for lipid droplet accumulation, hypoxia-inducible lipid droplet-associated gene expression, and altered lipid metabolism, with non-stem tumor cells in hypoxic zones exhibiting distinct metabolic and transcriptional signatures akin to GSCs, reinforcing the idea that organoids can effectively model how hypoxia orchestrates coordinated metabolic and phenotypic adaptation [173]. Profiling of GSC-derived GBM organoids revealed intrinsic immune-like transcriptional states, including upregulation of antigen presentation machinery, cytokine signaling, and interferon-response genes in specific malignant subpopulations, suggesting that tumor-intrinsic inflammatory programs can be modeled and perturbed in organoids [111].

Receptor–ligand inference tools such as CellChat and CellPhoneDB, which are leading computational tools for inferring cell–cell communication from scRNA-seq data, have been applied to long-term organoid ecosystems to generate hypotheses about which host cell types and ligands mediate plasticity-relevant crosstalk. In GLICO models, ligand–receptor modeling identified astrocytes as dominant hubs communicating with GSCs via pro-tumorigenic ligands such as FGF1 under hypoxic stress, with further validation using CellPhoneDB confirming that astrocytes interacted heavily with GSCs [161]. Similar analysis in cortical organoid invasion models by Ge et al. used CellChat v1.6.1 to reveal extensive bidirectional interactions between malignant cells and neurons, astrocytes, and oligodendrocyte lineage cells [120]. Together, these studies suggest that organoid-based systems can capture cell–cell interactions that push transitions toward invasive or therapy-resistant phenotypes. Beyond biochemical signals, ECM composition and mechanical constraints in TME profoundly influence GBM cell behavior and plasticity. Fan and colleagues have developed structured assemblies that couple GBM organoids with regionally specified forebrain organoids within 3D-printed microchannel devices used to mimic distinct neuron subtype environments and study how niche identity shapes invasion and growth patterns [174]. This organoid system revealed preferential GBM invasion into dorsal forebrain-like regions and niche-dependent transcriptional adaptations, effectively modeling how regional neuronal identity modulates tumor phenotypes. Microfluidic and hydrogel-based systems complement organoid models by offering precise control over ECM stiffness, composition, and flow while incorporating microglia and stromal cells. In a collagen-based microfluidic platform, GBM-conditioned media were shown to drive robust microglial activation and invasion that were largely insensitive to MMP inhibition or glycolytic blockade but strongly reduced by inhibition of Rho-associated protein kinase (ROCK) pathway. These findings imply protease-independent ECM remodeling as a key mechanism of microglial motility in GBM [175]. A related hydrogel system integrating brain-derived decellularized ECM with HA methacrylate created a composite matrix with GBM-like mechanical properties and biochemical composition, and co-culture with microglia increased GBM proliferation and invasion [148]. Together, these engineered organoid platforms provide mechanistic insight into how ECM and mechanics modulate GBM plasticity and can be integrated with organoid models to interrogate specific microenvironmental triggers.

### 4.3. Modeling Therapy-Induced Evolution in GBM Organoids

TMZ has been extensively evaluated in GBM organoid systems, revealing patient-specific and microenvironment-dependent resistance patterns. GSC-derived organoids subjected to TMZ alone or in combination with radiotherapy showed variable changes in size, proliferation, apoptosis, and senescence, with many organoids exhibiting only modest shrinkage but pronounced spatial remodeling of proliferative and quiescent niches [109]. In another study, PDGBOs treated with clinically relevant TMZ doses plus radiation displayed persistent proliferative rims and largely maintained organoid architecture, in contrast to more pronounced cytotoxicity in 2D and sphere cultures from the same patients [21]. Radiotherapy is another common treatment for GBM that has been found to contribute to therapy-induced evolution. Radiation studies in cerebral organoids combine DNA damage markers such as γH2AX (phosphorylation of Ser139 in histone H2AX) and 53BP1 (53-binding protein 1) along with analysis of differential gene expression post-radiotherapy to track the specific cells or states that radiation selects for [176,177]. GBM organoids have also provided evidence that spatially organized, phenotypically diverse niches contribute to therapy resistance and evolution. Hubert and colleagues exposed GSC-derived organoids to radiotherapy and determined that these organoids exhibited more heterogeneous and blunted responses than matched sphere cultures, with proliferative activity persisting in peripheral rims and quiescent, hypoxic cores showing relative survival. These patterns are consistent with niche-protected, stress-adapted subpopulations that can survive initial treatment and seed regrowth of the tumor [157]. Furthermore, Sundar and colleagues reported that GBM organoids maintained overall structure and viability and retained proliferative rims after combining TMZ and radiotherapy, whereas corresponding spheres showed more uniform cytotoxicity [109]. Both studies highlight the need for 3D spatial organizations to model TMZ- and radiotherapy-induced evolution in GBM and detail how organoids are crucial to fulfilling this requirement. Integration of radiation dose modeling alongside hypoxia-modulating interventions in organoids may further clarify how microenvironmental gradients modulate radiation-induced tumor evolution.

## 5. Technological Innovations Enabling Deeper Evolutionary and TME Interrogation in GBM Organoids

### 5.1. Spatial Transcriptomics Within Organoids

Spatial transcriptomics has become a cornerstone for connecting evolving cell states to 3D microenvironmental context because it preserves spatial relationships that are otherwise lost during dissociation-based single-cell profiling [178]. The foundation of array-based spatial transcriptomics is the capture of poly(A) RNA from intact tissue sections onto surfaces bearing spatially indexed oligonucleotides, followed by reverse transcription, library construction, sequencing, and computational reconstruction of where transcripts originated [179]. In organoid workflows, this typically requires careful control, most commonly fresh-frozen or lightly fixed samples embedded for cryosectioning, because RNA integrity, section adhesion, and permeabilization kinetics are strongly altered by organoid density and ECM content [180]. Methodologically, the basic spatial transcriptomics framework places thin sections onto barcoded capture arrays, generating transcriptome-wide maps at defined spot resolutions [181]. The development of Slide-seq extended this concept by transferring RNA from freshly frozen sections onto densely packed DNA-barcoded beads with bead positions determined by in situ sequencing, enabling near single-cell spatial mapping [182]. Slide-seqV2 subsequently improved sensitivity through modifications to bead synthesis, array indexing, and library preparation, while maintaining a resolution of 10 μm [183]. In parallel, imaging-based spatial transcriptomic approaches provide a complementary path to spatial resolution and gene multiplexing by performing iterative rounds of in situ hybridization and imaging [184]. One such model is called Multiplexed Error-Robust Fluorescence In Situ Hybridization (MERFISH), and it uses error-robust barcoding and sequential imaging to quantify and localize hundreds-to-thousands of RNA species at single-molecule resolution within single cells [185]. SeqFISH+ (sequential fluorescence in situ hybridization plus) further scaled this model toward sets capable of imaging up to 10,000 genes by controlling optical crowding through subset imaging and computational reconstruction of full expression profiles [186]. In organoid applications, these imaging-based spatial transcriptomics approaches are methodologically demanding, but they offer an unparalleled analysis of interactions relevant to GBM progression such as cytokine hotspots, tumor–microglia contact, or invasion-associated transcriptional programs [187].

A recurring technical issue in spatial transcriptomics is that spot-based capture arrays record mixtures of cell types within each spot, requiring heavy deconvolution and reference mapping to interpret niche composition [188]. Cell2location addresses this by using a Bayesian model to integrate single-cell reference profiles with spatial data, estimating cell-type abundances while accounting for technical effects and background [189]. Another model called Tangram provides an alternative deep-learning framework that aligns single-cell transcriptomes to spatial measurements, supporting transcriptome-wide reconstruction and mapping of cell states to tissue coordinates [190]. In one organoid application, Lin and Colleagues have used Cell2location to map cellular composition in PDGBOs and coupled Tangram with scRNA-seq data to create a map that matches cellular state with PDGBO structure [191]. In another organoid study, Albiach and colleagues used Tangram to measure gene expression across multiple PDGBOs and average it into one map displaying the gene expression profiles of PDGBO cellular components [192]. With further research and refinement, these computational strategies now show strong promise to become powerful analytical tools for GBM organoids [35]. Organoid-specific spatial transcriptomics has progressed beyond single-section snapshots towards 3D mapping, which is especially relevant for evolutionary modeling where selection pressures vary spatially. Ishahak and colleagues have engineered GBM organoid platforms that have explicitly combined single-cell and spatial transcriptomics to examine how subtype-specific oncogenic programs reorganize cell states and spatial architecture within organoid microenvironments [18]. Lozachmeur and colleagues described a strategy using consecutive sections and double-barcoded arrays to generate three-dimensional reconstructions of gene-expression heterogeneity within human cerebral organoids [193]. This is especially important since spatial transcriptomics can serve as a benchmark for organoid reproducibility by quantifying whether organoids accurately recreate spatial gene-regulatory programs and tissue-like patterning, rather than relying solely on cellular composition [194]. Furthermore, the development of neural organoid spatial profiling, including recent work by Yang et al. mapping distinct spatial patterning and transcriptomic landscapes in human neural organoids, suggests a new avenue for advancing the study of GBM in organoids [195].

### 5.2. Organoid-on-a-Chip Systems

**Figure 3:**
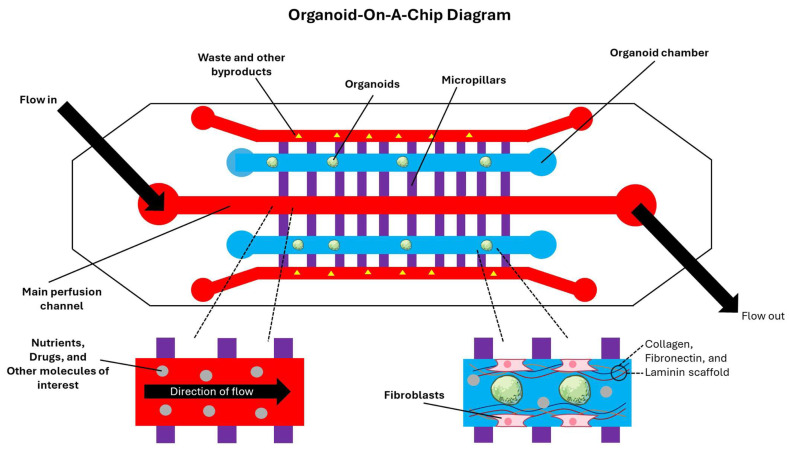
A fundamental framework for the design of an organoid-on-a-chip model. The main perfusion channel enables the flow of blood, nutrients, drugs, and other molecules of interest. Once in the main perfusion channel, the perfusable materials can diffuse through the micropillars into the organoid chamber, where the organoids are grown. In addition to receiving the perfusable material from the main perfusion channel, the organoid chamber can contain ECM components like fibroblasts and collagen that simulate the TME of GBM, promoting organoid growth in a physiological relevant environment. Variables like the flow rate and specific perfusable materials can be controlled, making the organoid-on-a-chip model a highly tunable structure to assess and grow GBM organoids.

While spatial transcriptomics offers high-resolution snapshots of niche structures and state distributions, TME-driven evolution is inherently dynamic. As such, organoid-on-a-chip systems are therefore increasingly used to impose controlled gradients and time-varying selection pressures [196]. Organoid-on-a-chip platforms apply microfluidic engineering to organoids to create controlled, perfused microenvironments that approximate better the dynamic gradients such as oxygen and nutrient availability and mechanical cues that shape GBM evolution [197]. These devices typically combine defined tissue chambers that encapsulate organoids or spheroids within ECM-like hydrogels, perfusion channels that continuously deliver media and circulating immune and vascular components, and micropillars that enable diffusion coupling between flow and tissue compartments [198]. In brain organoid-on-chip applications, soft lithography-based polydimethylsiloxane devices have been fabricated with parallel culture chambers and central perfusion channels interconnected by micropillar structures, enabling continuous medium flow while maintaining 3D tissue organization [199]. Perfusion is not merely a passive element of TME. It is a driving force that can materially alter organoid viability and spatial physiology by reducing necrotic cores and stabilizing gradients, which in turn change the evolutionary landscape [200]. In the context of GBM modeling, microfluidic systems provide a route to impose defined oxygen gradients that emulate hypoxia-driven selection, a key driver of invasiveness through pro-mesenchymal transitions [201]. Additionally, advancements in microfluidic platforms have integrated oxygen control with long-term live microscopy for 3D tissues, enabling sustained imaging under controlled hypoxic conditions rather than relying on discrete measurements [202]. Emerging microfluidic designs further extend this by generating programmable dissolved oxygen gradients, supporting experiments where spatially structured hypoxia can be treated as an experimental variable shaping invasion and treatment resistance [203]. All the fundamental features of the organoid-on-a-chip model in a miniature form are easily tunable and readily usable by the researchers in finding answers to the critical questions about the TME of GBM without the investment of huge resources and time (Figure 3).

A major advance for TME fidelity has been the incorporation of vascular and BBB features into chip-based GBM models, which is central for studying perivascular niches, immune trafficking, and drug delivery constraints [204]. Straehla and colleagues have described a vascularized GBM spheroid model in a microfluidic device that includes self-assembled endothelial networks with astrocytes and pericytes, enabling interrogation of transport across BBB-like vasculature into tumor compartments [134]. Additionally, Silvani and colleagues developed a hybrid microfluidic-bioprinting approach that produced the compartmentalized vascularized GBM spheroid-on-a-chip systems designed to emulate both a functional BBB and an adjacent 3D perivascular tumor niche under controlled stress and cell–ECM mechanical interactions [205]. These microfluidic platforms operationalize selective pressures, a key principle for evolutionary studies, and allow these pressures to be tuned reproducibly [206]. Recent GBM-focused demonstrations include microgravity-cultured PDGBOs integrated with microfluidic chips to evaluate CAR T cell efficacy, utilizing dynamic microenvironments to study immune-mediated evolutionary bottlenecks [207]. Organoid-on-a-chip presents the opportunity to integrate immune components, vascular flow, and biophysical cues within a single platform to effectively model the features of the GBM microenvironment that drive therapy escape, invasiveness, and immune evasion [208]. One limitation is that device materials and fluidics can distort drug exposure [209]. Polydimethylsiloxane, a common material for microfluidic devices, can absorb small hydrophobic molecules and shift effective doses, altering dose–response relationships unless mitigated by coatings or alternative materials [210]. As organoid-on-chip platforms increasingly aim to model selection under clinically relevant pharmacokinetics, careful construction of microfluidic devices and their standardization become as important as the development of organoids [211].

### 5.3. Advanced Imaging and Live-Cell Lineage Tracking

Even with dynamic gradients and perfusion, mechanistic understanding of GBM evolution in organoids depends on tracking changes like invasion roadways, clonal competition, and fate changes over extended periods of time, necessitating advanced imaging and live-cell lineage tracking. Advanced imaging methods provide the temporal and spatial resolution needed to observe evolutionary behaviors in organoid-based microenvironments [212]. In GLICO models, Linkous and colleagues have demonstrated that tumor cells can be fluorescently labeled with green fluorescent protein (GFP) to enable longitudinal monitoring of invasion patterns and dynamic phenotypes such as tumor microtube formation [19]. They used stable GFP reporters and two-photon imaging to quantify tumor volume and visualize microtube networks within cerebral organoid tissue. Two-photon microscopy is particularly valuable for organoids because it provides deeper penetration with reduced photobleaching compared with conventional confocal imaging, supporting repeated imaging of thick tissues [213]. Imaging becomes substantially more powerful for evolutionary inference when paired with lineage tracking because it allows observed behaviors and phenotypes to be traced longitudinally [214]. Recent organoid lineage technologies include the use of fluorescent reporter-based lineage tracing in cortical organoids to track the development of neural progenitors [215]. Lentiviral barcoding strategies, such as CellTagging, provide a scalable route to label many cells with unique barcodes that can later be read out by scRNA-seq, enabling reconstruction of lineage development and fate flights [216]. The clustered regularly interspaced short palindromic repeats (CRISPR)-based lineage recording further enables barcoding and recording of lineage relationships over time, which is immensely valuable in cerebral organoids where selective pressures vary spatially and temporally [217]. Furthermore, recent work by Gentile and colleagues describe the iTracer system, which combines reporter barcodes with inducible CRISPR–Cas9 perturbations to longitudinally analyze clonal development and lineages in cerebral organoids [218].

In addition to measuring clonal dynamics, advanced imaging and live tracking can be used to study GBM migration and invasion. Goranci-Buzhala and colleagues have described a rapid invasion assay of GBM in cerebral organoids, providing a controllable framework for comparing invasive capacity and testing perturbations that modulate infiltration. Furthermore, they coupled this with time-lapse imaging to determine invasion speed, directional persistence, and invasion-associated morphology [22]. A limitation in organoid imaging is optical scattering, which constrains depth and resolution in live imaging and complicates accurate reconstruction [219]. Tissue clearing protocols address this by improving imaging depth, enabling whole-organoid immunolabeling and 3D microscopy [220]. Dekkers and colleagues provided a detailed approach for high-resolution 3D imaging of fixed and cleared organoids, including steps for fixation, immunostaining, clearing, and imaging across entire organoid volumes [221]. Another imaging modality called the light-sheet fluorescence microscopy has been shown to image large cleared organoid samples rapidly with lower photobleaching, making it well-suited for reconstructing invasion networks and spatial niche structure [222]. Protocol developments that combine expanded clearing and light-sheet fluorescence microscopy further enhance the ability to map fine neural architectures at the single-cell level [223]. Advanced imaging in GBM organoids is moving beyond structure and lineage toward functional phenotyping. Morelli and colleagues have used NAD(P)H fluorescence lifetime imaging microscopy in GBM organoids to predict early TMZ response on a timescale of roughly one week after surgery, illustrating how metabolic imaging can report treatment-induced state change without extensive disruption of the original tumor architecture [224]. Reynolds and colleagues have recently introduced live organoid cyclic imaging, a bioorthogonal click chemistry strategy (click chemistry reactions occurring in biological environments with no obstacle to normal cellular functions) in which tetrazine-based quenching extinguishes fluorescence between cycles in seconds and enables repeated stain–image–quench rounds in PDGBOs while preserving viability [225]. In practice, the limiting step then shifts from image acquisition to analysis, because live imaging requires automated segmentation and tracking pipelines to convert raw images into reliable lineage trees and quantifiable phenotypes [226]. Taken together, this field is moving toward integrated workflows that combine low-phototoxicity live imaging, whole-volume endpoint reconstruction, and molecular lineage recording. These multimodal designs are likely to be essential for elucidating invasion, clonal selection, and evolutionary escape in GBM organoid models.

### 5.4. Genome Editing for Evolutionary Interrogation in Organoids

**Figure 4:**
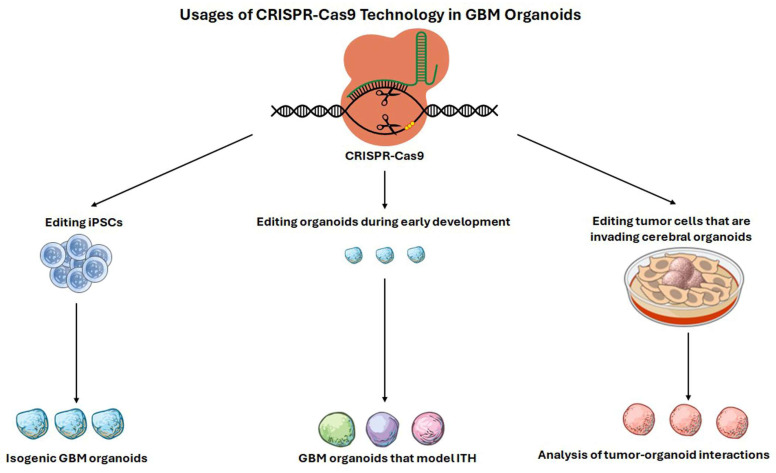
The three main applications of CRISPR-Cas9 in GBM organoids. First, editing iPSCs enables control of organoid lineage, allowing for the creation of isogenic organoids. This allows for the creation of standardized organoids, supporting reproducible findings. Second, editing organoids in their early developmental stages enables mosaic checking, producing heterogeneity within individual organoids. This effectively models the ITH seen in GBM. Third, editing tumor cells that are invading cerebral organoids supports investigations into how specific mutations can drive GBM invasion and expansion.

CRISPR-based engineering enables mechanistic dissection of evolutionary dynamics in organoids by allowing defined mutations to be introduced, combined, or timed to test how TME selection shapes tumor trails [227]. Organoid genome editing can occur at multiple levels: editing iPSCs before organoid formation to generate isogenic backgrounds, mosaic editing during early organoid development to emulate ITH, or editing tumor cells introduced into brain organoids to assess interactions with the host-like TME (Figure 4) [228]. The choice among these strategies determines whether the experimental model reflects early tumor initiation within a developing tissue-like context or later-stage invasion and adaptation in a structured niche [229]. Bian and colleagues have introduced genetically engineered cerebral organoids as a platform for modeling tumorigenesis, demonstrating that the introduction of oncogenic mutations can drive GBM growth and associated programs within cerebral organoids [230]. Complementary approaches inserted oncogenic drivers into specific locations to generate neoplastic cerebral organoids and track tumor initiation and progression [231]. These studies illustrate key practical components of evolutionary organoid engineering: single guide RNA selection and validation, delivery, clonal expansion, and multi-level quality control. More recent work has moved toward detailed profiles that highlight how specific genotypes reshape spatial organization and cell-state landscapes, critical for modeling TME-driven evolution. Wang and colleagues developed laboratory-engineered GBM-like organoids (LEGOs) using CRISPR–Cas9-engineered loss of GBM-relevant tumor suppressors followed by comprehensive profiling to determine the effect of genetic heterogeneity in GBM on clonal evolution and the efficacy of handpicked therapeutics [232].

In parallel, engineered GBM organoids have been developed using specific oncogenic mutation combinations, with single-cell and spatial transcriptomics used to resolve how these drivers disrupt regulatory programs and reorganize spatial architecture [18]. These platforms allow controlled comparisons of isogenic or near-isogenic organoids differing in defined pathways, enabling tests of how microenvironmental gradients preferentially select for various transcriptional programs [233]. Perturbation approaches are also expanding from single-gene edits to multiplex strategies, which can broadly assess evolutionary hypotheses [234]. Despite these advances, genome editing in organoids introduces many confounders that must be explicitly controlled [235]. Further, off-target effects and clonal bottlenecks during the selection process can create engineered artifacts that masquerade as microenvironmental selection if proper controls are not included [236,237]. Moreover, alternative perturbation methods such as short hairpin RNA (shRNA)-mediated depletion of tumor suppressors in organoids can offer complementary routes to engineer GBM-like phenotypes but may differ in knockdown completeness and stability relative to CRISPR edits [238].

### 5.5. Artificial Intelligence, Machine Learning, and Multimodal Integration with Organoids

AI and machine learning (ML) methods are increasingly used to convert organoid experiments into quantitative, scalable, and ultimately predictive platforms for TME and evolutionary studies [239]. The first wave of organoid-focused ML targeted image-based phenotyping, where deep learning can segment organoids, track growth, and quantify invasion margins without fluorescent labels [240]. Matthews and colleagues have developed a model called OrganoID that provides a deep-learning-based approach for organoid analysis and tracking that enables high-throughput extraction of size and shape metrics from standard microscopy [241]. The Organoid Brightfield Identification-based Therapy Screening (OrBITS) model introduced by Deben et al. similarly emphasizes automated analysis of longitudinal brightfield imaging for organoids, supporting scalable drug screening and kinetic response profiling without complex staining workflows [242]. In neural organoid contexts, Metzger and colleagues used deep-learning-supported computational pipelines to extract phenotypes from patterned or structured organoid systems, enabling systematic quantification of morphological outcomes that would be challenging to score manually [243]. ML approaches are beginning to explicitly address drug-response prediction by integrating organoid molecular data [244]. PharmaFormer, which is a prediction model for clinical drug response based on custom transformer design and transfer learning, uses a transformer-based architecture and transfer learning across cell line and organoid pharmacogenomic datasets to predict clinical outcomes from drug and molecular inputs, illustrating how organoid experiments can feed into clinically relevant predictive modeling [245].

Since organoid studies frequently generate combinations of scRNA-seq, spatial transcriptomics, and imaging, AI and ML can enable multimodal integration [246]. The Multi-Omics Factor Analysis (MOFA) framework introduced factor-based unsupervised integration for multi-omic datasets, supporting decomposition of heterogeneity into interpretable latent dimensions [247]. The subsequent MOFA+ model provides a statistical framework for integrating multimodal single-cell data via latent factor modeling and variational inference, enabling identification of variation across complex designs [248]. TotalVI extends this logic to paired RNA and protein single-cell measurements by probabilistic modeling of biological signals, yielding latent spaces useful for integrating immune phenotypes with transcriptional programs [249]. Deep generative modeling has become particularly important for organoid evolutionary questions because these models can both correct batch effects and learn latent representations that support inference and prediction [250]. Lopez and colleagues have introduced the scVI model, which uses deep generative modeling for single-cell transcriptomics to enable probabilistic representations of cell states and scalable integration across batches [251]. The scGen model demonstrated how generative models can be used for perturbation prediction in single-cell data, providing a conceptual route to forecasting state shifts under therapies or microenvironmental stresses [252]. The scArches model further supports transfer learning and reference mapping, which is highly relevant when organoid datasets must be aligned to patient tumors or spatial reference frameworks to interpret evolutionary relevance [253]. These methods are operationalized through scVI-tools, which provide a unified system for probabilistic modeling across multiple single-cell modalities in complex studies [254]. Taken together, AI and ML methods are enabling a shift from descriptive phenotyping to quantitatively integrated multi-modal systems in which morphology, spatial context, lineage, and molecular states can be jointly modeled to predict evolutionary transitions and therapeutic outcomes, highlighting promising applications for using in GBM organoids [255].

## 6. Limitations and Challenges Associated with GBM Organoids

### 6.1. Limitations of Current GBM Organoids for Modeling the TME and Evolutionary Dynamics

A major limitation of current GBM organoids is that they incompletely reproduce the cellular ecology that shapes tumor evolution in patients [41]. Spatial and single-cell studies of primary GBM have established that macrophages, microglia, endothelial cells, pericytes, reactive astrocytes, and infiltrating lymphocytes are not passive bystanders, but active determinants of hypoxia tolerance, invasion, vessel remodeling, and treatment resistance [256]. In particular, the identification of perinecrotic hypoxia-associated macrophages that destabilize tumor vasculature through adrenomedullin signaling illustrates how tightly vascular and immune architecture are coupled in GBM, and how difficult these interactions are to reproduce in tumor-only organoids [80]. Even when stromal or immune populations are initially retained from fresh surgical tissue, they are often variably represented and may diminish over time in culture, which means that late-passage metrics can become progressively less dependable [257]. Watanabe and colleagues have further shown that some ‘immune-like’ signatures can be GBM-intrinsic and present even in organoids lacking immune cells, which are highly informative biologically but also a caution against equating immune transcriptional programs with a reconstructed immune microenvironment [111]. Another limitation is that most GBM organoid systems reproduce hypoxia only because of diffusion constraints rather than as a vascularized and perfused niche with realistic hemodynamics, endothelial signaling, and BBB behavior [157]. This problem is exacerbated in organoids with a diameter greater than 500 μm because the vasculature cannot adequately perfuse the entire organoid, leading to additional artificial hypoxia in the tumor core [258,259]. This distinction matters because the hypoxic core seen in avascular organoids is not necessarily equivalent to the perinecrotic, macrophage-rich, vessel-remodeling niche observed in patient tumors, where hypoxia coexists with abnormal angiogenesis and immune recruitment [260]. The same issue applies to perivascular niches, which in vivo regulate stemness, invasion, and treatment response, but are only partially approximated in most organoid platforms unless endothelial or stromal compartments are deliberately engineered into the system [261]. Efforts such as the vascularized GBM tumoroid described by Tatla et al. begin to address this gap by incorporating vasculature-related stromal components and hypoxia-responsive angiogenic behavior [127].

**Figure 5:**
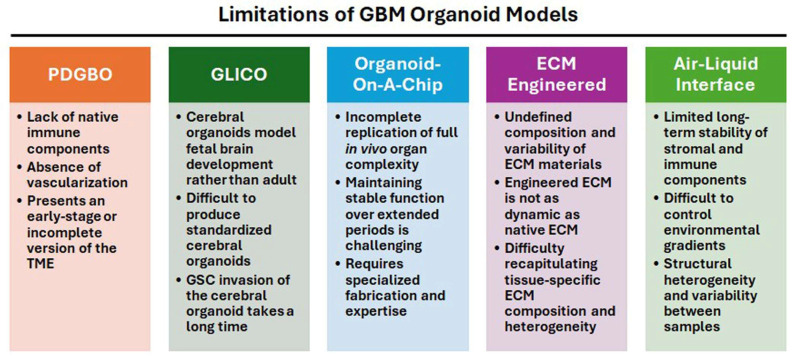
The limitations of specific GBM organoid models. Despite their advantages, each of these models has its own set of limitations. Current research has addressed some of the limitations associated with GBM organoids; however, there are certain limitations that continue to persist.

Current organoids also incompletely model evolutionary dynamics because the relevant evolutionary state in GBM is often a plastic cell state embedded in a niche, rather than a genetically fixed clone acting in isolation [262]. Longitudinal analyses of matched primary and recurrent GBM now suggest that recurrence is frequently driven by state switching and microenvironmental remodeling as much as by genetic selection, meaning that any model of evolution must preserve both transcriptional heterogeneity and the extrinsic cues that stabilize or redirect it [172]. PDGBOs can preserve ITH and extrinsic signaling, and organoid studies suggest that they can also serve as longitudinal patient avatars [107]. While organoid models are especially useful for studying tumor initiation and heterogeneity, they do not recapitulate the full evolutionary history of a patient’s tumor that has already undergone immune sculpting, microenvironmental adaptation, and therapy exposure. GLICO models partly compensate for these deficiencies by placing GBM cells into a human neural context that is otherwise absent from tumor-only organoids, but they introduce a separate set of limitations. In GLICO, patient-derived GSCs home toward and invade cerebral organoids. The resulting organoids form tumors that resemble patient material, making the model particularly valuable for studying invasion and tumor–neural contact [19]. Goranci-Buzhala and colleagues expanded GLICO by establishing rapid invasion assays that could quantify invasion, integration, interaction with mature neurons, and responses to perturbation [22]. Fan and colleagues advanced this concept by assembling dorsal or ventral forebrain organoids that showed region-specific effects on GBM invasion and neural activity [174]. However, these GLICO models remain developmentally immature relative to the adult human brain and still lack full immune, vascular, and ECM complexity [110]. As such, they model select tumor–brain interactions rather than the developed adult. For this reason, the current role of organoids should be complementary models that isolate particular ecological axes of GBM and not standalone replicas of the full tumor ecosystem. The known limitations of five well-studied GBM organoid models are presented as a snapshot (Figure 5).

### 6.2. Standardizing GBM Organoid Workflows for Reproducibility

Since the deductions drawn from GBM organoids depend heavily on how the model was generated, reproducibility in this field begins before culture and extends beyond the protocol, highlighting how standardization remains a major limitation with organoid models. Reproducible organoid models treat tissue acquisition, model class, time in culture, and assay architecture as explicit experimental variables. Jacob and colleagues provided one of the clearest methodologic frameworks by generating GBM organoids directly from surgically resected tissue without dissociation in chemically defined medium, passaging and cryopreserving the resulting organoids, and defining a clinically relevant timescale of roughly 2–4 weeks for organoids establishment and 5–7 days for CAR T co-culture experiments [104]. By extension of these protocol papers and the broader organoid-standardization literature, future studies should explicitly report the tumor region sampled, the interval from resection to processing, transport conditions, and whether the tissue was kept intact or dissociated, because these are plausible sources of variability between models even when the culture is nominally identical. PDGBOs, GSC-derived organoids, GLICO, ECM-engineered organoids, and immune-competent assembloids each preserve different compartments, and each therefore requires different quality-control criteria and different expectations about what is reproducible. Specifically, PDGBO studies should report whether organoid establishment was performed with intact tumor pieces or after dissociation, whether the medium was fully chemically defined, how passaging and cryopreservation were performed, and the exact day or passage at which phenotyping or drug testing began [104]. GSC-derived organoids should report whether exogenous mitogens were used continuously because Watanabe and colleagues have argued that growth-factor-rich conditions can bias clonal representation and therefore alter the evolutionary paths visible in culture [111]. GLICO studies should state the developmental stage and regional identity of the recipient organoid and how long tumor–brain contact was allowed to proceed before analysis [174]. This level of standardized detail is required since invasion phenotypes are strongly dependent on host maturity and architecture. Immune-enabled models should additionally specify whether immune cells were autologous or allogeneic, whether matched peripheral blood mononuclear cells were used, the duration of co-culture, and the functional outputs chosen to quantify response, such as cytolysis, cytokine release, single-cell transcriptional shifts, or T cell receptor expansion [121].

Reproducibility also depends on benchmarking organoids against the source tumor rather than relying on morphology alone. Jacob and colleagues reported that their GBM organoids recapitulated histologic features, cellular diversity, gene expression, and mutational profiles of matched parental tumors, thereby establishing a baseline expectation that organoid fidelity should be measured across multiple data types rather than inferred from 3D growth alone [107]. Golebiewska and colleagues have extended this logic by showing that PDGBOs of primary and recurrent gliomas preserve histopathologic, genetic, epigenetic, and transcriptomic characteristics, which is particularly important for studies of evolutionary dynamics [263]. LeBlanc and colleagues have similarly shown that patient-derived explants are genetically similar to parent tumors and retain their transcriptional heterogeneity, underscoring both the promise and the incompleteness of ex vivo models as representations of the original lesion [264]. Verduin and colleagues have reported retention of major driver mutations, copy-number alterations, MGMT promoter methylation, and treatment sensitivity in patient-derived GBM organoids, supporting the case for routine genomic benchmarking when organoids are proposed as translational avatars [21]. Collectively, these studies argue that minimum metrics for GBM organoids should include histopathology, key genomic lesions, epigenetic status, and cell-state composition assessed by single-cell or comparable profiling with repeated benchmarking rather than a single validation at establishment, emphasizing that tracking an organoid’s development over time is an important consideration.

Organoid analytics require a level of standardization no less than culture conditions do, since similar organoids can yield quite different conclusions depending on how invasion, viability, or cell-state composition are quantified. Invasion studies already illustrate this point. Goranci-Buzhala and colleagues emphasized assay versatility for invasion and neuron interaction, whereas Fan and colleagues integrated region-specific organoid assembly with transcriptional analysis and electrophysiologic phenotyping, broadening the meaning of an invasion assay beyond simple distance measurement [22,174]. Immunotherapy studies provide an even stronger case for comprehensive organoid analytics since CAR T and checkpoint blockade responses can be missed or misinterpreted if one measures only viability while ignoring antigen loss, cytokine release, T cell expansion, and remodeling of cell–cell interactions. Logun and colleagues linked organoid cytolysis and cytokine profiles to match patient CAR T metrics, while Baisiwala and colleagues used single-cell analysis and TCR sequencing to show pembrolizumab-driven expansion of patient-specific CD4 clonotypes, demonstrating that standardized reproducible inference depended on thorough multi-modal analysis [106,121]. Standardization should not be confused with homogenization. The aim is not to force all GBM organoids to look alike, but to separate true patient-specific variation from variation introduced by specimen handling, culture bias, and analysis choices. That distinction is especially important for evolutionary studies, where the most interesting signal may be preserved divergence between organoids derived from different regions, time points, or treatment states of the same disease.

### 6.3. Translating TME- and Evolution-Informed GBM Organoid Findings to Clinical Practice

The translational potential of GBM organoids lies in their ability to function as patient-specific, experimentally tractable tumor ecosystems rather than as mere culture surrogates for bulk tumor cells. This matters because clinically meaningful questions in GBM increasingly concern ecological and evolutionary behavior, such as why some tumors shift toward mesenchymal states after therapy, why some lesions remain immunologically cold, and why invasion or recurrence occurs in distinct ways despite similar driver mutations. PDGBOs are attractive in this setting because they can be generated on a timeframe that begins to intersect with clinical decision-making, and early studies have already shown that mutational profiles and drug responses can be linked within the same organoid platform [107]. Golebiewska and colleagues strengthened the translational case by generating organoid and xenograft avatars from both primary and recurrent gliomas, thereby moving beyond static disease modeling toward a framework for studying longitudinal tumor evolution and resistance [263]. Verduin and colleagues likewise have shown that patient-derived GBM organoids can preserve heterogeneity while reflecting treatment sensitivity, supporting their use as functional complements to histopathology and molecular diagnostics [21]. Organoid systems also offer a powerful route for translating insights about microenvironmental control of tumor state and resistance, which is essential if GBM treatment is to move beyond static target selection. In HOTT models, Ge and colleagues showed that perturbing the neural microenvironment by knocking down PTPRZ1 shifted co-cultured patient tumors toward a mesenchymal state and increased tumor microtube length, thereby demonstrating that clinically relevant tumor phenotypes could be driven by extrinsic cues originating outside the malignant compartment [120]. Watanabe and colleagues similarly found that patient-derived organoids could recapitulate glioma-intrinsic immune programs and therapy-resistant progenitor populations, suggesting that organoids might be especially valuable for separating tumor-cell-autonomous programs from those that would truly require immune-cell participation [111]. Wang and colleagues used CRISPR-engineered GBM-like organoids and multi-omic profiling to map coordinated molecular networks and identify genotype-associated drug sensitivities [232]. These organoid platforms are most compelling when they are used to evaluate specific hypotheses about evolutionary plasticity rather than as generic screening tools separated from tumor ecology.

Even so, organoid predictive value should not be confused with full clinical substitution. Standard GBM organoids do not model systemic pharmacokinetics, corticosteroid exposure, BBB-restricted distribution, radiation field geometry, or the prolonged immunologic feedback loops that occur in patients undergoing multimodal therapy, so any response measured in organoids should be interpreted as one component of clinical sensitivity rather than a stand-alone treatment recommendation. This limitation is especially relevant for anti-angiogenic, macrophage-targeted, and invasion-directed therapies, where treatment outcome depends on vascular function, myeloid dynamics, and region-specific brain architecture that are only partially reconstructed in most current organoid formats. The logical translational path, therefore, is not to replace clinical or molecular assessment with organoids, but to integrate organoids with pathology, single-cell and spatial profiling of the parent tumor, and longitudinal clinical data from recurrence and response. Prospective studies should evaluate whether organoid phenotypes correlate with variables like progression-free survival, recurrence patterns, and therapy-induced state transitions. Only those correlations will establish whether organoids add any useful information beyond what is captured by current diagnostics. If that standard can be met, GBM organoids might be positioned not simply as preclinical models, but as controllable models of how the TME and evolutionary dynamics shape patient-specific therapeutic vulnerability.

## 7. Conclusions and Future Directions

Despite recent advancements, GBM remains a fatal malignant disease in which near-universal recurrence after maximal surgical resection and standard chemoradiation reflects tumor-intrinsic genetic diversity and microenvironment-enabled adaptation under therapy [265]. Longitudinal genomic studies of matched primary and recurrent GBMs demonstrate that treatment itself can reshape clonal architecture and accelerate the emergence of resistant lineages, including courses consistent with TMZ-driven mutations and selection for therapy-resistant clones [266]. At the cellular level, single-cell profiling has reinforced that GBM is composed of multiple, interconverting malignant states whose proportions and behaviors are influenced by niche cues and external stressors, providing a mechanistic basis for rapid phenotypic switching during treatment [267]. Together, these observations underscore why preclinical platforms must recapitulate both ITH and TME features that condition evolution rather than focusing solely on isolated tumor cell populations.

Organoid technologies have become central to this effort because they enable the controlled reconstruction of human-relevant microenvironments while preserving clinically meaningful aspects of GBM architecture and diversity. PDGBOs showed that 3D growth can preserve hypoxic gradients and cancer stem cell heterogeneity, two hallmarks tightly linked to therapeutic resistance and spatially patterned evolution [157]. In parallel, GLICO models have provided a compelling tumor-in-brain context, in which patient-derived GBM cells infiltrate human neural tissue and form invasive architectures that closely resemble patient tumors [19]. The most compelling advances point toward organoid ecosystems that move beyond tumor-only cultures to explicitly model TME-driven state changes and therapeutic escape. HOTT systems provide a framework to interrogate how extrinsic microenvironment cues reshape GBM cellular composition and behavior while also enabling exploration of the impacts of perturbations on the tumor and TME [120]. Efforts to incorporate additional TME features, such as vasculature and immune components, are rapidly expanding the scope of organoid modeling to explore therapeutic opportunities. Vascularized tumoroid systems have been used to study GBM angiogenesis in the context of hypoxia-related cues, enabling controlled tests of how vascular niches and growth factor environments shape tumor phenotypes relevant to therapy response [127]. iHOTT allows for interrogation of patient-matched immune infiltration, immune–tumor signaling, and immunotherapy responsiveness within a brain-like environment, including the capacity to observe checkpoint inhibitor–associated immune shifts and T cell clonal dynamics [121]. When coupled with single-cell transcriptomics, organoid invasion assays additionally allow investigators to map how GBM cells shift transcriptional programs after interaction with neural microenvironments, linking observable invasion phenotypes to state transitions that may forecast treatment resistance [160]. GLICO models have extended this by demonstrating that organoid ecosystems can recapitulate malignant cell-state diversity while revealing intercellular transfer phenomena, offering new routes by which GBM may reprogram surrounding brain-like cells and reinforce resistant niches [159]. Multi-omic atlases of engineered GBM organoids have begun to map how genetic heterogeneity propagates into functional heterogeneity across transcriptomic, metabolomic, and proteomic levels [232].

Together, these approaches describe a future in which organoids may serve as a common experimental model for integrating lineage tracing, spatial profiling, functional screening, and therapy-mimetic selection into one comprehensive model of GBM. Although organoid models have not been established in clinical practice, a promising futuristic direction is the development of patient-specific GBM “evolutionary avatars” that integrate serial tumor sampling with perfused vascular networks, patient-matched immune and stromal compartments, and tunable ECM architectures within microfluidic platforms [268,269]. In such systems, real-time imaging, biosensing, and lineage recording can be combined with single-cell and spatial multi-omic readouts to track how GBM adapts during therapy rather than only before and after treatment [270]. Coupling these next-generation organoids to perturbation screens and computational modeling may ultimately enable prospective prediction of recurrence pathways, identification of microenvironment-dependent vulnerabilities, and selection of individualized therapeutic combinations that more faithfully anticipate the dynamic biology of GBM in patients. Looking forward, the impact and reproducibility of GBM organoid models will depend on sustained improvements in standardization, benchmarking against matched patient data, and the systematic incorporation of missing TME components, such as perfused vasculature, accurate ECM architectures, and patient-relevant immune and stromal populations. Equally important will be designing experiments that mirror the realities of treatment to ensure that organoid-observed evolutionary trajectories remain faithful to the selective pressures that shape recurrence in patients. With further adoption and refinement, organoids might find a role in clinical practice as not just a proxy for GBM biology, but a practical engine for discovering microenvironment-conditioned resistance mechanisms and enabling faster, more individualized translation from patient tumor biology to therapeutic strategy.

## Figures and Tables

**Table 1:** Cell components in TME of GBM and their impact on GBM survival.

Cell Type	Model	Effect on GBM Survival	References
TAMs	scRNA-seq of mice GBM and patient-derived GBM spheroids	TAMs actively reprogram GBM cells toward a proliferative mesenchymal state in both in vivo and in vitro models. TAMs secrete Oncostatin M, which activates the signal transducer and activator of transcription 3 (STAT3) pathway in GBM cells. STAT3 activation is associated with tumor cell survival, growth, and immune evasion.	[34]
TAMs	GL261 cell culture and mice GBM	TAMs in both the in vivo and in vitro chemoresistant GBM models displayed greater immunosuppressive effects than TAMs non-chemoresistant GBM models and supported GBM proliferation.	[58]
Astrocytes	C57BL/6 mice implanted with GL261 cells	Astrocytes in the tumor core and periphery were ineffectively to regulate excess glutamate due to under expression of glutamate transporter-1 (GLT-1), which promotes neuronal death. Astrocytes were also implicated in the upregulation of transglutaminase 2 (TGM2), an enzyme that is important to tumor extracellular matrix (ECM) development and growth.	[59]
Astrocytes	Astrocytes co-cultured with tumor samples from patients with GBM	These astrocytes showed increased expression of the JAK/STAT pathway. Inhibition of the JAK/STAT pathway with Ruxolitinib resulted in less tumor growth and a more favorable immune environment.	[60]
Astrocytes	Astrocytes co-cultured with U87MG cells to make GBM spheroid	Astrocytes form Connexin (Cx)43 gap junctions with GBM cells. After formation of the Cx43 gap junctions, GBM cells displayed greater growth and invasiveness. In Cx43 knockout GBM spheroids, there is a significant decrease in tumor invasiveness.	[61]
Pericytes	C57BL/6 mice implanted with patient-derived GSCs	Inhibition of pericytes with ibrutinib weakened the blood-tumor-barrier by disrupting the tight junction proteins that hold it together. Furthermore, this contributed to a weaker tumor vasculature and improved drug delivery of chemotherapeutics.	[62]
Pericytes	GBM spheroids co-cultured with pericytes	GBM spheroids were treated with temozolomide (TMZ), a chemotherapeutic. The GBM spheroids that were co-cultured with pericytes demonstrated significantly greater drug resistance and survival than the GBM spheroids without a pericyte co-culture. Specifically, chemokine (C-C motif) ligand 5 (CCL5) is upregulated in response to TMZ.	[63]
Endothelial Cells	LN229 GBM cells co-cultured with endothelial cells and NOD-SCID mice implanted with patient-derived GBM samples	Endothelial cells release stromal cell-derived factor-1 alpha (SDF-1α), which promotes GBM cells to transition to a GSC-like state. Specifically, SDF-1α promotes the upregulation of the transcription factor glioma-associated oncogene homologue 1 (GLI1).	[64]
GSCs	hESC-derived GBM organoids	Overexpression of the transcription factor mesenchyme homeobox protein 2 (MEOX2) in GSCs. Organoids with the overexpression of MEOX2 in GSCs displayed stronger tumor growth and TMZ resistance compared to organoids with a MEOX2 knockout.	[65]
GSCs	Patient-derived GSC spheroids and C57BL/6 mice implanted with patient-derived GSCs	GSCs heavily produce ATP-binding cassette 4 (ABCB4) transporters that can pump out intracellular drugs. Furthermore, GSCs export ABCB4 through exosomes to differentiated GBM cells, spreading drug resistance. ABCB4 knockdown models displayed weaker TMZ resistance than control models.	[66]
GSCs	Individually grown GBM and GSC cell lines	The GSC cell lines displayed greater quiescent phenotypes compared to the GBM cell lines. After the cell lines were exposed to radiation, the GBM cell lines displayed a shift in their metabolic prolife. However, the quiescent GSC cell lines maintained their metabolic prolife, highlighting GSC resistance to radiotherapy.	[67]

TAMs = Tumor associated macrophages, GSCs = Glioblastoma stem cells, GBM = Glioblastoma multiforme, and ECM = Extracellular matrix.

**Table 2:** Recent organoid models and their roles in modeling GBM.

Organoid Model	GBM Source	Special Features	Results	References
GBM organoid with native ECM synthesis	JX6 and U251MG cell lines	Endogenously generated ECM without the use of Matrigel	Organoids reached more than 4 mm and exhibited a size-dependent shift from homogeneous aggregates to tissue-like, tumor-relevant transcriptional programs with strong upregulation of ECM pathway. Organoids displayed stiffness in the range reported for diffuse gliomas.	[137]
PDGBO	Patient-derived GSCs	None	The organoids formed a connected network containing both tumor microtubes and tunneling nanotubes, enabling intracellular mitochondrial transfer. The GSC populations showed differences in mitochondrial transfer efficiency and heterogeneous gene expression.	[138]
GLICO	Patient-derived GBM cells	Co-culture with three different organoids: forebrain, midbrain, and spinal cord	Single-cell analyses showed a consistent shift toward neuron/glia progenitor-like states after engraftment in all three organoids, demonstrating that patterned brain organoids can model region-specific neural inputs that shape GBM invasion.	[139]
Patient derived gliosarcoma organoid	Patient-derived gliosarcoma cells	None	Organoids were generated without enzymatic dissociation, preserving parental tumor histology, genomic alterations, and cell populations. They demonstrated infiltrative behavior, and scRNA-seq revealed mesenchymal-dominant programs, highlighting aggressive GBM biology.	[140]
PDGBO with ECM	Patient-derived GSCs	The PDGBOs were grown in an exogenous ECM of gelatin methacryloyl and hyaluronic acid methacryloyl	A gelatin methacryloyl–hyaluronic acid (HA) methacryloyl ECM supported formation of compact GBM organoids while preserving GSC characteristics and proliferative capacity. Compared to Matrigel–HA methacryloyl, the gelatin methacryloyl–HA methacryloyl ECM produced more compact clones and greater proliferation.	[141]
PDGBO with ECM and endothelial cells	Patient-derived GSCs	Encapsulation in an engineered ECM and co-cultured with endothelial cells	Adding endothelial cells enhanced perivascular niches, producing robust CD31 expression consistent with endothelial integration, tumor stemness, proliferation, and hypoxic signaling. Endothelial co-culture also increased multilineage marker expression, reflecting GBM plasticity and heterogeneity.	[142]
PDGBO with endothelial cells, pericytes, and astrocytes	Patient-derived GSCs	Vascular development due to co-culture with endothelial cells and pericytes	Organoids displayed quiescence and niche-mediated protection from chemotherapy, modeling clinically relevant therapy resistance. Comparative transcriptomics showed that glia–vascular contact induced gene programs linked to immune suppression, mirroring adaptations seen in vivo.	[143]
GLICO	Patient-derived GSCs	None	Pine et al. profiled chromatin accessibility in 28,040 single cells from five patient-derived GSC lines to capture tumor cell states. The data revealed TME-driven, dynamic chromatin remodeling programs that accompany transitions between GBM cell states.	[144]
PDGBO	Patient-derived GBM cells	None	Darrigues et al. screened anti-invasive compounds in PDGBOs, measuring invasion from organoid margins. While tubulin inhibitors showed strong invasion inhibition in the cell line model, responses in PDGBOs were highly variable, reflecting ITH typical of GBM.	[145]
GLICO	Patient-derived GBM cells	None	GBM cells formed hallmark tumor microtubes, established neuron–glioma synapses and generated an interconnected network with coordinated Ca^2+^ signaling, paralleling features observed in patients. scRNA-seq showed that GBM cells retained heterogeneous transcriptional programs and reproduced clinically relevant therapy-resistance patterns.	[146]
GBM organoid with intact BBB	U87MG cell line	Organoids were composed of six different cells: U87MG, microglia, oligodendrocytes, neurons, pericytes, and endothelial cells	GBM organoids formed an intact BBB with endothelial surfaces and tight-junction borders that excluded fluorescent dextran, reproducing the selective permeability that limits chemotherapy delivery. Fluorescent ultrasmall gold nanoparticles carrying doxorubicin penetrated and distributed throughout organoids.	[147]
GBM organoid with ECM and microglia co-culture	U87MG cell line	Encapsulation in hyaluronic acid methacryloyl and decellularized brain-derived ECM; organoids were also co-cultured with HMC3 cells to include microglia	The researchers observed that adding microglia increased invasion distance by nearly 40% with higher expression of ECM-remodeling genes. Microglia co-culture also enhanced proliferation and the system supported >90% viability with spindle-like, interconnected GBM morphologies.	[148]

GBM = Glioblastoma multiforme, PDGBO = Patient-derived glioblastoma organoid, GLICO = Glioma cerebral organoid, BBB = Blood–brain barrier, GSCs = GBM stem cells, ITH = Intratumoral heterogeneity, ECM = Extracellular matrix, HMC3 = Human microglial clone 3.

## Data Availability

No new data were created or analyzed in this study. Data sharing is not applicable to this article.
